# On the Relative Importance of Different Factors Explaining Health Plan Choices: Evidence From Mandatory Health Insurance in Switzerland

**DOI:** 10.3389/frhs.2022.847486

**Published:** 2022-04-15

**Authors:** Yanmei Liu, Stefan Boes

**Affiliations:** Department of Health Sciences and Medicine and Center for Health, Policy and Economics, University of Lucerne, Lucerne, Switzerland

**Keywords:** health plan choice, mandatory health insurance, socioeconomic characteristics, behavioral theories, personality traits, dominance analysis

## Abstract

Many factors influence health plan choices. Classical individual-level determinants include socioeconomic and health-related characteristics, and risk attitudes. However, little is known to what extent personality traits can determine insurance choices. Using representative survey data from Switzerland, we investigate the associations between choices of health plans and traditional individual factors as well as personality traits. We employ dominance analysis to explore the relative importance of the different predictors. We find that personality traits play an at least equally important role in predicting health plan choices as common factors like age, health status, and income. Our results have implications regarding recent efforts to empower people in making better health plan choices and support theoretical models that integrate insights from behavioral sciences.

## Introduction

In choice-based health insurance systems, like Switzerland, the Netherlands, Germany, or the marketplaces under the Affordable Care Act in the U.S., individuals must make decisions on their health plans. Depending on the system design, health plans can differ in terms of the insurers, deductibles (or other elements of cost-sharing), services covered, or restrictions in the access to certain health care providers. In this study, we focus on the health insurance system in Switzerland (1) and three dimensions of health plan choice, namely the deductible level, basic free-choice-of-provider plans vs. restricted-access plans, and supplementary hospital insurance. The Swiss setting offers an interesting case to study insurance decision-making due to these different choice dimensions and the related complexity of selecting a suitable plan. Understanding the factors that affect decision-making is vital for policymakers to design an insurance system that empowers people to make more informed choices. For instance, identifying the factors that are most important in plan selection may help design and adopt policies that target more simplified health plans, reflecting individual needs, and promote public education on the health insurance system.

Extensive theoretical and empirical work demonstrates that characteristics of the consumers, such as socioeconomic and demographic background, health-related factors, and risk preferences, influence decision-making for health plans [see (2, 3) for a review]. Expected utility theory as a benchmark addresses how risk-averse individuals make decisions to reduce uncertainty and maximize expected utility under budget constraints [see, e.g. (4, 5)]. Under this theory, income, risk preferences, and expected losses due to illness are key determinants of coverage decisions. Relatedly, state-dependent theory highlights that an individual's state, such as health or socioeconomic status, affects the individual's expected utility, which further influences the magnitude of risk aversion and insurance choice (3). In addition, through health and health care consumption, lifestyle factors have been conjectured to affect the selection of health plans using a structural equation model (6).

In this study, we aim to investigate another possible domain of determinants of health plan choices. Building on the literature seeking to enrich economic models of decision-making with insights from personality psychology [see, for example (7–11)], we specifically explore the role of personality traits in addition to the classical determinants mentioned above. A search in common databases like PubMed, Scopus, or Web of Science indicates that research on personality traits as determinants of health plan choices is largely underdeveloped. By the time we wrote this paper, such studies had not been conducted in Switzerland, and we found only two studies internationally that add personality traits as explanatory variables in models for health plan choices. One study from Germany finds that personality traits, and extraversion, in particular, are positively associated with the decision to opt out of statutory health insurance and to choose private insurance coverage (12). A second study for India reports similar results on uptake of health insurance, although the system and context are very different and the sample relatively specific (13). Whether personality traits can also predict health plan choices in the Swiss mandatory health insurance system, however, is still unclear.

Personality traits often refer to “the relatively enduring patterns of thoughts, feelings, and behaviors that reflect the tendency to respond in certain ways under certain circumstances” (14). The Big Five Inventory is often used to measure personality traits (15). The five broad trait dimensions or domains consist of openness to experience, conscientiousness, extraversion, agreeableness, and neuroticism. Openness to experience describes the appreciation of artistic and aesthetic experiences as well as the tendency to have imagination and creative ideas. Conscientiousness refers to the tendency to do a thorough job, being efficient and diligent. Extraversion reflects a person's characteristics of being outgoing, sociable, and talkative. Agreeableness refers to warmth, trust, and generosity. Neuroticism captures emotional (in)stability such as anger, worries, and anxiety (16, 17). Each of the Big Five domains describes another aspect of an individual's personality and, as such, can affect the individual's preferences, actions, and behaviors, which may all relate to health plan choices. For example, conscientiousness could relate to the individual's efforts searching for information on suitable health plans and deciding based on personal needs and circumstances. Extraversion could relate to alternative information-seeking channels, and agreeableness, or rather its absence, to the person's decision-making power and the ability to make financially complex decisions.

Using data from the Swiss Household Panel (SHP), we aim to gain deeper insights into this topic. First, we employ logistic regression models to investigate the association between choices of health plans and four sets of determinants representing an individual's socioeconomic, demographic, and health-related characteristics, attitude toward risk-taking, and personality traits. Second, we apply dominance analysis to determine the relative importance of the predictors in the logistic regressions. The hypothesis is that apart from the traditional individual characteristics, personality traits are essential predictors of choices of health plans, which is confirmed in our analysis.

The next section briefly describes the institutional background relevant to our study. Section data and methods provides an overview of the data source, the variables, and descriptive statistics, and it outlines the methods. Section results shows the main findings of the logistic regressions and the dominance analysis, which are then discussed in section discussion. The final section concludes our study.

## Institutional Background

Basic health insurance in Switzerland is compulsory for all and provided by about 60 private insurers. It covers the essential statutory benefits for illness, maternity, and accidents (as long as not covered by accident insurance) and has been in place since 1996 with the Swiss Health Insurance Act. Every insured person can freely choose the insurer and pays a community-rated premium, which is independent of income and individual risk but varies by cantonal premium regions, three age groups, type of health plan, and provider. There are six annual deductible options (CHF 300, 500, 1,000, 1,500, 2,000, and 2,500) for adults. The minimum level is the standard deductible, and the other options are voluntary. Individuals with voluntary deductibles receive premium rebates, limited to 70% of the incremental deductible amount (1). In addition, the insureds pay a 10% coinsurance up to CHF 700 per year once the medical expenses reach the selected deductible level.

The insureds can choose between unrestricted and restricted access plans. While the standard insurance model offers unrestricted access to health care providers across the residential canton, alternative health plans may restrict this choice and require a general practitioner or a medical call center to act as a gatekeeper. Alternative plans are commonly referred to as regulated managed care plans, including health maintenance organization (HMO) plans, telemedicine models, and preferred provider organization (PPO) health plans. The standard plan corresponds to the highest premium, whereas alternative plans offer premium rebates. Alternative plans may also provide partly or fully waived deductible and coinsurance rates, although the latter waiver rarely happens (1). Voluntary deductibles can be integrated into managed care plans, and the overall premium rebate amounts to 50% relative to the standard health plan in any case (1). Low-income households have the right to receive premium subsidies, which are regulated by the cantons (18).

In addition to basic insurance, consumers can purchase supplementary insurance to cover additional risks or improve coverage. One type of supplementary insurance is private hospital insurance, which ensures the free choice of a hospital or a specialist/head physician, and a private or semi-private hospital ward [see (1) for more details on the Swiss health insurance system].

## Data and Methods

The 2015 and 2017 waves of the Swiss Household Panel (SHP) are used for this study. The SHP is a nationally representative sample of Swiss households managed by the Swiss Centre of Expertise in the Social Sciences (FORS); see https://forscenter.ch/projects/swiss-household-panel/ and (19) for details. Only adults aged 26 and over are included in this study because young adults (18–25 years) and children (0–17 years) face different insurance conditions. In addition to demographic, socioeconomic, and health-related characteristics, the dataset contains information on attitudes toward taking risks, the Big Five personality traits, and health insurance. We chose a cross-sectional design since personality traits are considered stable over time. We confined the insurance data to the 2017 wave, which is the first wave this information was collected and closest to the time point when the Big Five Inventory was surveyed in the panel (in 2015), in order to reduce issues of panel attrition, giving us a final sample of 8,093 individuals.

An overview of all variables included in the study is presented in Table 1.

**Table 1:** Definitions of variables.

**Variable**	**Definition**
**Outcomes: Health plan choice**	
Voluntary deductible	Annual deductible in two categories: 1 for voluntary deductible: CHF 500, 1,000, 1,500, 2,000, 2,500 0 for standard deductible: CHF 300
Alternative plan	1 for an alternative plan (managed care), 0 for standard basic plan
Supplementary insurance	1 for supplementary hospital insurance, 0 no supplementary hospital insurance
**Explanatory variables**	
**Socioeconomic and demographic characteristics**	
Female	1 for females, 0 for males
Age	Divided into five age groups: 26–35, 36–45, 46–55, 56–65, 66+
Education	Highest educational attainment in 3 categories: low, intermediate, and high level
Log income	Log of equivalized yearly net household income
Married	1 for married, 0 for not married
Urban	Typology of Community: urban vs. rural
**Health-related factors**	
Good health	Self-rated health status: 1 for good health, 0 for fair or poor health
Chronic	1 for chronic health conditions, 0 no chronic illness
Doctor visits	Doctor consultations in the year preceding the date of the interview: 1 for yes, 0 for no
Hospital stays	Hospital stays in the year preceding the date of the interview: 1 for yes, 0 for no
**Risk attitude**	
Risk	Risk level rated on an 11-point scale, grouped into five categories: 1 for risk-averse, 2 moderately risk-averse, 3 risk-neutral, 4 moderately risk-seeking, 5 risk-seeking
**Personality traits**	
	Measures how a person responds to “I see myself as someone who”:
Openness	is original, comes up with new idea; values artistic, aesthetic experiences; has an active imagination
Conscientiousness	does a thorough job; tends to be lazy; does thing efficiently
Extraversion	is talkative; is outgoing, sociable; is reserved
Agreeableness	is sometimes rude to others; has a forgiving nature; is considerate and kind to almost everyone
Neuroticism	worries a lot; gets nervous easily; remains calm in tense situations

*Source: Swiss Household Panel (SHP); for further information about the data, see (17)*.

### Outcome Measures

There are three outcome measures. The choice of a deductible is essential for all insurance policies in the Swiss system, so the first outcome variable describes whether an individual opts for the standard or a voluntary higher deductible. The second outcome relates to the type of health plan, denoting restricted vs. unrestricted access to healthcare providers, i.e., the choice between an alternative (managed care) plan and the standard health plan. The third outcome variable is also dichotomous and defined as whether or not the individual holds supplementary hospital insurance.

### Explanatory Variables

The independent variables are grouped into four categories (see Table 1): socioeconomic and demographic characteristics, health-related factors, risk attitudes, and personality traits.

### Socioeconomic and Demographic Characteristics

Socioeconomic and demographic indicators include age, sex, educational attainment, income, civil status, and residential region. Age refers to the age at the time of the interview in the following categories: 26–35, 36–45, 46–55, 56–65, 66+. Educational attainment refers to the highest educational level achieved. It is coded in three categories according to the International Standard Classification of Education (ISCED): low (ISCED 1, 2), intermediate (ISCED 3), and high level (ISCED 4, 5, 6). The variable for civil status equals one for being married and zero for not being married, and it is included due to its relationship with health trajectories (20). The log-transformed equivalized yearly net household income is used to measure financial resources. The equivalized household income is adjusted for household size and composition and converted into per capita income using the modified OECD equivalence scale (17). The net income is obtained by subtracting social security contributions from gross income, but without deducting taxes or insurance premiums (21). Finally, we control for the regional settings by differentiating between urban and rural environments.

### Health-Related Factors

The health-related variables refer to health status and health care needs. The health status consists of two elements. The first one is subjective health which refers to a person's self-assessed overall health status, and the second indicates the presence of a chronic health condition. Subjective health is measured on a scale from one (very well) to five (not well at all). We aggregated this information into a binary variable indicating if the person is in good health (values 1, 2) or fair or poor health (values 3, 4, 5). Chronic health conditions are measured dichotomous, indicating whether or not an individual has a long-term illness or health problem.

In addition, utilization of health care services from the previous survey year is used as a proxy for health care needs, which can also reflect a person's health conditions. Two variables are used: whether the person visited at least once a physician (general practitioners or specialists but excluding dentists) and whether the person stayed in a hospital in the year preceding the interview.

### Risk Attitudes and Personality Traits

The SHP measures attitudes toward taking risks in general on an 11-point scale ranging from 0 = “avoid taking risks” to 10 = “fully prepared to take risks” (17). In our analysis, we classified this information into five categories, defined as risk-averse (value 0), moderately risk-averse (1–3), risk-neutral (4–5), moderately risk-seeking (6–7), and risk-seeking (8–10).

In the SHP wave 17 in 2015, personality traits were assessed with the 15-item Big Five Inventory (BFI-15) (17). The BFI-15 is an established and validated self-report measure that consists of three items for each of the five personality dimensions (17, 22). The 15 items are statements followed by “I see myself as someone who…”. Each item is measured on an 11-point response scale ranging from 0 = “disagree strongly” to 10 = “agree strongly” (17, 22). Following previous literature, we applied confirmatory factor analysis (CFA) to develop constructs for each personality trait from the original items included in the survey, which were then used as explanatory variables in the logistic regression models and the dominance analyses (23). While the CFA yielded meaningful constructs for the personality traits openness, agreeableness, conscientiousness, and neuroticism, for the trait extraversion, the CFA showed convergence problems in obtaining the factor loadings. For this reason, we computed an average value of the relevant three items for this trait.

### Descriptive Statistics

Table 2 displays summary statistics for the predictor variables, by health plan choices for the three choice dimensions presented above. Overall, the majority of respondents select voluntary deductibles and managed care plans but no supplementary hospital insurance.

**Table 2:** Descriptive statistics.

**Predictors**	**Deductibles**		**Plan type**		**Private hospital insurance**	
	**Standard deductible**	**Voluntary deductibles**	**Standard plan**	**Alternative plans**	**Basic insurance**	**Supplementary insurance**
Female	0.602	0.497	0.551	0.543	0.544	0.549
**Age**						
26–35 years	0.068	0.164	0.100	0.142	0.146	0.083
36–45 years	0.099	0.184	0.117	0.162	0.164	0.106
46–55 years	0.195	0.254	0.230	0.228	0.238	0.212
56–65 years	0.222	0.198	0.218	0.200	0.197	0.229
66+	0.417	0.200	0.334	0.268	0.254	0.371
**Education**						
Low	0.170	0.088	0.145	0.112	0.142	0.095
Intermediate	0.564	0.526	0.508	0.558	0.555	0.509
High	0.266	0.386	0.347	0.330	0.303	0.396
Log income	10.976 (0.502)	11.103 (0.481)	11.067 (0.558)	11.038 (0.468)	10.959 (0.466)	11.216 (0.520)
Married	0.619	0.622	0.582	0.638	0.603	0.653
Urban	0.733	0.721	0.754	0.707	0.696	0.775
Good health	0.761	0.890	0.814	0.850	0.831	0.846
Chronic	0.539	0.299	0.433	0.372	0.388	0.405
Doctor visits	0.883	0.702	0.786	0.763	0.751	0.815
Hospital stays	0.216	0.122	0.166	0.158	0.157	0.175
**Risk attitude**						
Risk-averse	0.102	0.045	0.073	0.067	0.077	0.061
Moderately risk-averse	0.167	0.154	0.150	0.157	0.158	0.150
Risk-neutral	0.347	0.323	0.333	0.335	0.331	0.338
Moderately risk-seeking	0.246	0.312	0.277	0.290	0.281	0.289
Risk-seeking	0.138	0.167	0.166	0.151	0.153	0.162
**Personality traits**						
Openness	6.387 (1.355)	6.356 (1.338)	6.415 (1.340)	6.371 (1.363)	6.374 (1.358)	6.400 (1.344)
Conscientiousness	4.483 (0.703)	4.488 (0.640)	4.459 (0.701)	4.498 (0.650)	4.473 (0.669)	4.506 (0.674)
Extraversion	5.054 (1.403)	5.203 (1.321)	5.047 (1.346)	5.180 (1.378)	5.138 (1.376)	5.119 (1.351)
Agreeableness	−4.917 (0.749)	−4.864 (0.701)	−4.876 (0.742)	−4.895 (0.715)	−4.890 (0.735)	−4.889 (0.703)
Neuroticism	0.247 (1.116)	0.103 (1.120)	0.233 (1.136)	0.124 (1.100)	0.160 (1.117)	0.174 (1.106)
Number of observations	2,856	4,435	2,779	5,091	5,220	2,770

*Source: Swiss Household Panel (SHP), own calculations.*

*Sample proportions and means are reported for the categorical and continuous variables, respectively. Standard deviations are presented in parentheses only for the continuous variables. The analysis is based on a total sample of 8,093 individuals. Sample sizes between outcome columns somewhat differ due to missing data in the outcomes*.

With respect to the socioeconomic and demographic background of the respondents, more men than women have voluntary deductibles, whereas there are only minor gender differences in managed care plans and private hospital insurance. Concerning age, younger individuals are more likely to choose voluntary deductibles and restricted-access plans than the older age groups, but they are less likely to subscribe to supplementary hospital insurance. The more educated are more likely to choose voluntary deductibles and private hospital insurance, while individuals with an intermediate education level are most likely to have a managed care insurance model. As income increases, people have a higher chance to opt for a higher deductible and private hospital insurance. Married people and those living in rural environments are more likely to have an alternative plan with gatekeeping, whereas individuals who are married and living in urban settings are more likely to have supplementary hospital insurance. However, there are only minor differences in marital status and urban-rural environment regarding deductible choice.

Among the health-related characteristics, respondents who rate their health status better or do not have a chronic health condition are more likely to have voluntary deductibles or an alternative health plan. Relatedly, respondents who used physician services less likely or did not have inpatient stays in the previous year are more likely to have plans with higher deductibles or access restrictions. On the other hand, those with poorer health status and with higher health care needs are more likely to have supplementary hospital insurance, as one would expect.

Regarding risk attitude, respondents who report being more willing to take risks tend to favor health plans with voluntary deductibles and managed care plans. They are also more likely to have supplementary hospital insurance. As for personality traits, there are only minor variations observed between the targeted and baseline health plans. If anything at all, the descriptive statistics indicate that individuals who score high on conscientiousness, extraversion, and agreeableness, and low on openness and neuroticism tend to prefer a voluntary deductible to the standard deductible. While people who report high conscientiousness and extraversion, and low openness, agreeableness, and neuroticism are more likely to choose a managed care plan, those who are low in extraversion but high in the other four traits are more likely to hold supplementary hospital insurance.

### Methods

We utilize logistic regression models to explore the role of different predictors in choosing health plans, namely voluntary vs. standard deductibles, alternative insurance models vs. the basic model, and supplementary private hospital insurance vs. basic insurance. In doing so, we proceed in two steps. First, we estimate and compare the odds ratios for all predictor variables described in section explanatory variables. The odds ratios describe how much, in the fitted models, the probability of choosing a specific type of health plan changes relative to the probability of selecting the reference plan with a unit change in the predictor for which the odds ratio is calculated (24).

However, the logistic regression coefficients, and odds ratios derived from them, do not provide a good measure of the relative importance of the predictors included in the model (25). One reason is that the relative magnitude of the regression coefficients depends on the scaling of the variables, and even if standardized, they are model-dependent and thus do not preserve their order when the model is changed. Moreover, if some regressors are correlated, then their standardized regression coefficients are typically smaller, and they may turn out statistically insignificant in the model, even though each of the regressors alone may still be important in predicting the outcome of interest (25–29). Therefore, in a second step, we use dominance analysis, one of the most frequently applied approaches to assess predictor importance (30–32) and particularly useful in situations like ours, where multiple, correlated factors such as psychological characteristics (33), are involved. Dominance analysis was initially developed for linear regression models (34) and later extended to logistic regressions (25) and hierarchical linear models (35).

There is no universal definition of importance, but, in general, importance is referred to as the contribution that a regressor makes in predicting a response variable according to specified criteria (34, 36). Dominance analysis determines predictor importance by accounting for a predictor's direct effect (i.e., when considered alone), its total effect (i.e., conditional on all other predictors), and its partial effect (i.e., conditional on all possible subsets of predictors) (25, 34). One predictor is considered more important than another if it contributes more than the other variable to the estimation of an outcome variable in every subset model, where only one predictor of the pair is included (36). In other words, a particular predictor is more important than another when its contribution is greater in the full model (as measured by the regression coefficients), on its own (as measured by zero-order associations with the outcome), and in every subset model (25). Thus, when correlated predictors do not show statistical significance in the logistic regression models, they still can be important to the model fit as measured with dominance analysis.

Dominance analysis computes three types of dominance: complete importance, conditional importance, and general importance (36). If the additional contribution of one predictor is greater than that of another predictor across all possible subset models, then the former predictor is completely dominant over the latter (36). If the average additional contribution within each model is greater for one predictor than the other, then the first predictor is said to dominate the second conditionally (36). If the average conditional contribution of one predictor is greater than that of another in all subset models, then the first is said to dominate the second generally (25).

While in linear regression models, a predictor's additional contribution can be measured as changes in *R*^2^, analogs of *R*^2^ must be used in logistic models (25). Different measures were discussed previously according to defined criteria as suitable analogs (25, 37–40). In this paper, we focus on McFadden's *R*^2^ to measure model fit as it satisfies all necessary criteria of an *R*^2^ analog (25).

## Results

### Logistic Regression Models

**Figure 1:**
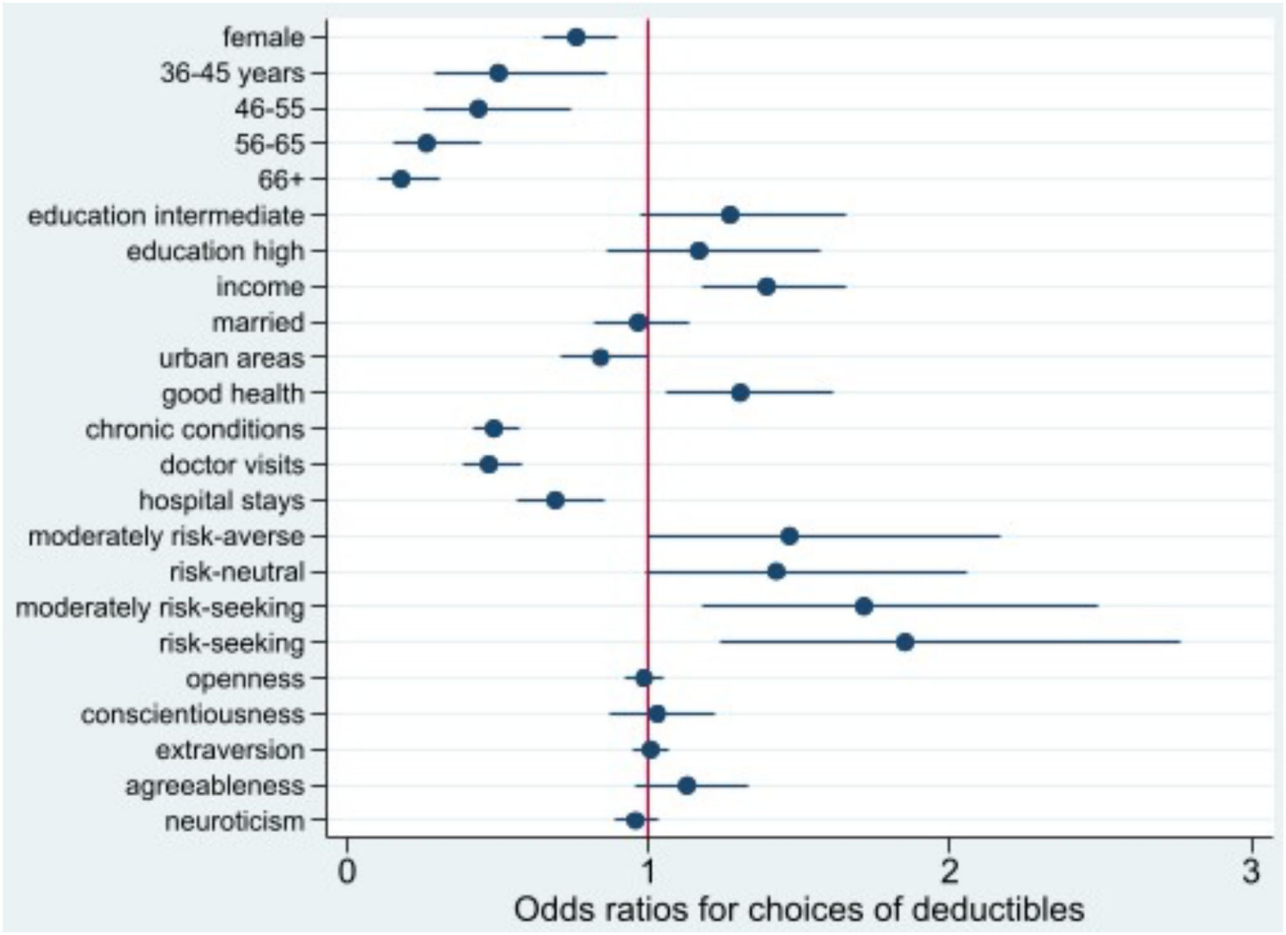
Odds ratio plot for choice of voluntary deductibles. Swiss Household Panel (SHP), own calculations. The figure shows the odds ratios from logistic regression models for the choice of voluntary deductibles, including 95% confidence intervals.

**Figure 2:**
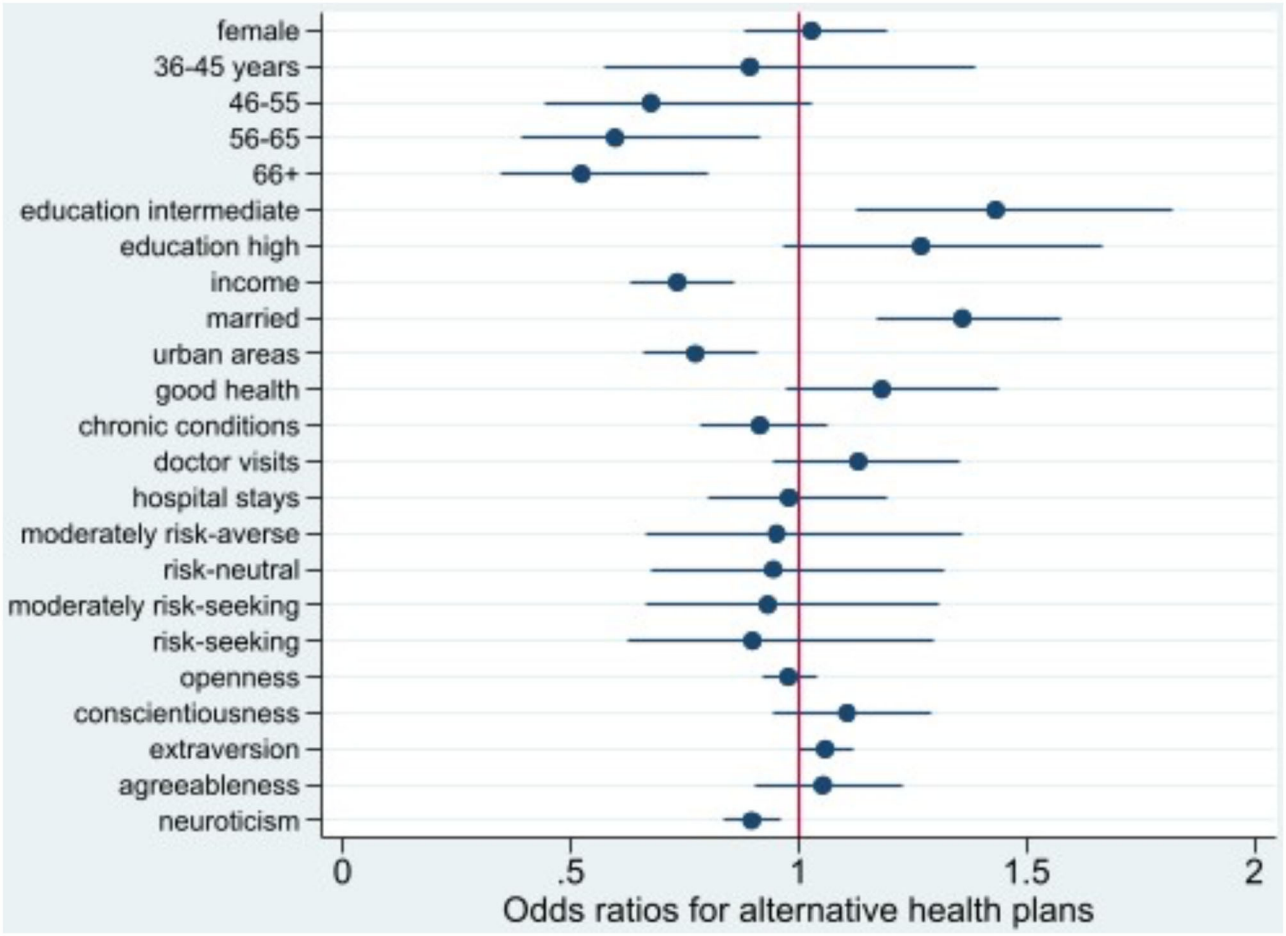
Odds ratio plot for choice of alternative health plans. Swiss Household Panel (SHP), own calculations. The figure shows the odds ratios from logistic regression models for the choice of an alternative health plan, including 95% confidence intervals.

**Figure 3:**
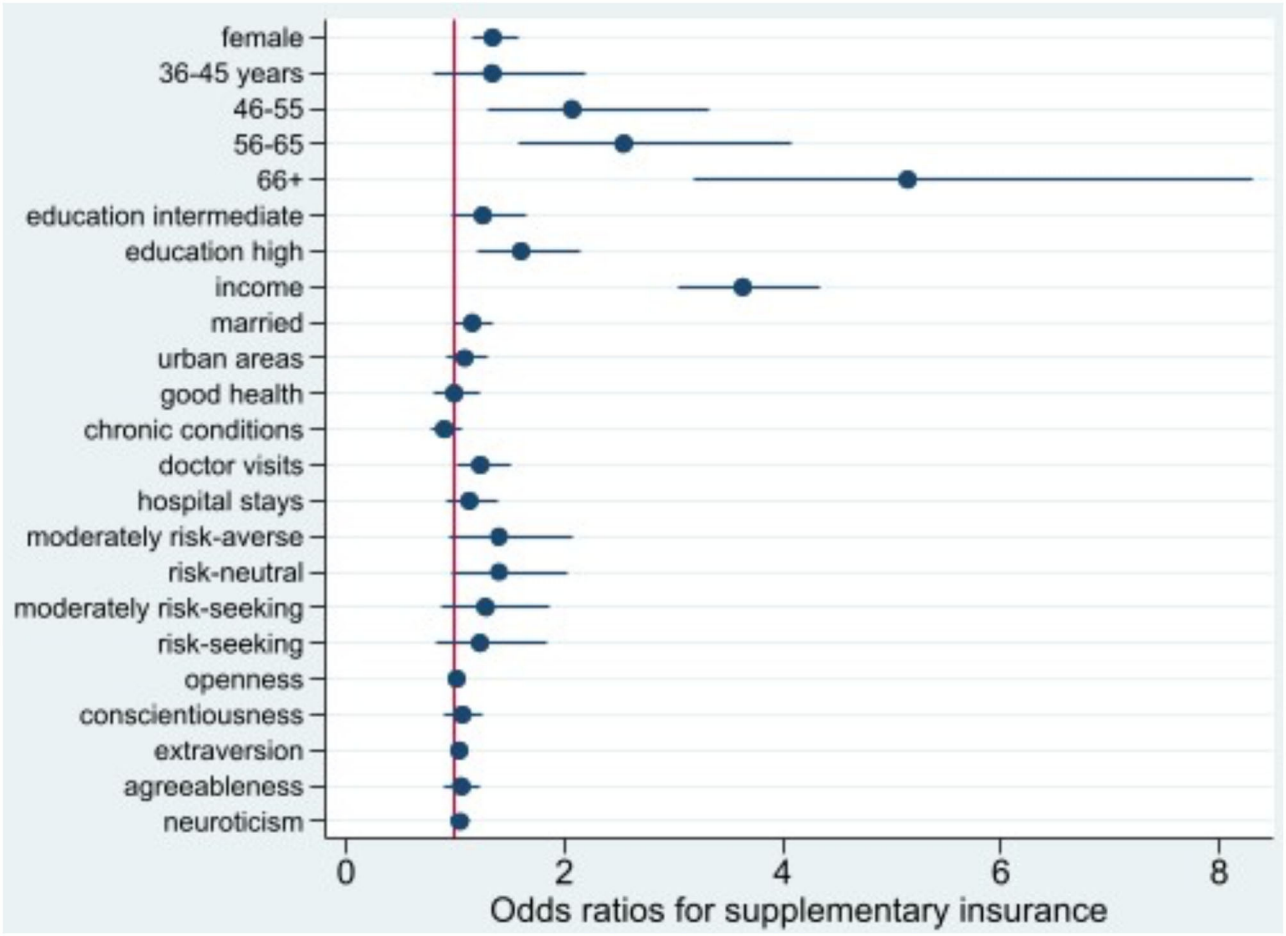
Odds ratio plot for choice of supplementary hospital insurance. Swiss Household Panel (SHP), own calculations. The figure shows the odds ratios from logistic regression models for the choice of supplementary hospital insurance, including 95% confidence intervals.

Odds ratios (OR) of the three logistic regression models are shown in Figures 1–3 (complete regression results are shown in Supplementary Table A1). Women (OR 0.76, *p* < 0.01) are less likely to opt for voluntary deductibles than men. Compared with the age group 26–35, the odds of selecting voluntary deductibles are smaller across all older age groups, and the odds decrease as age increases. While high-income earners, individuals in good health, and with a preference for taking more risks have a higher chance of purchasing voluntary deductibles, those with chronic conditions, doctor visits, or hospital stays are more likely to choose the standard deductible.

Individuals aged 56+ are less likely to opt for managed care plans with access restrictions than those aged 26–35. A similar pattern is observed for those with a higher income, living in urban settings, and exhibiting higher levels of neuroticism. However, married people, persons with an intermediate education and higher levels of extraversion tend to choose alternative insurance models.

For women, individuals with higher education, and those who consulted doctors in the past year, the odds of purchasing supplementary insurance are larger than the odds for their baseline groups. The odds of purchasing private hospital insurance increases significantly with income (OR 3.64, *p* < 0.001) and age, especially for those aged 66 years and over (OR 5.15, *p* < 0.001).

### Dominance Analysis

We computed complete dominance, conditional dominance, and general dominance for all three outcomes of interest, and the results of the three types of dominance statistics were consistent. For ease of exposition, we only present the general dominance weights since they are the most frequently reported metric from dominance analysis (30).

**Figure 4:**
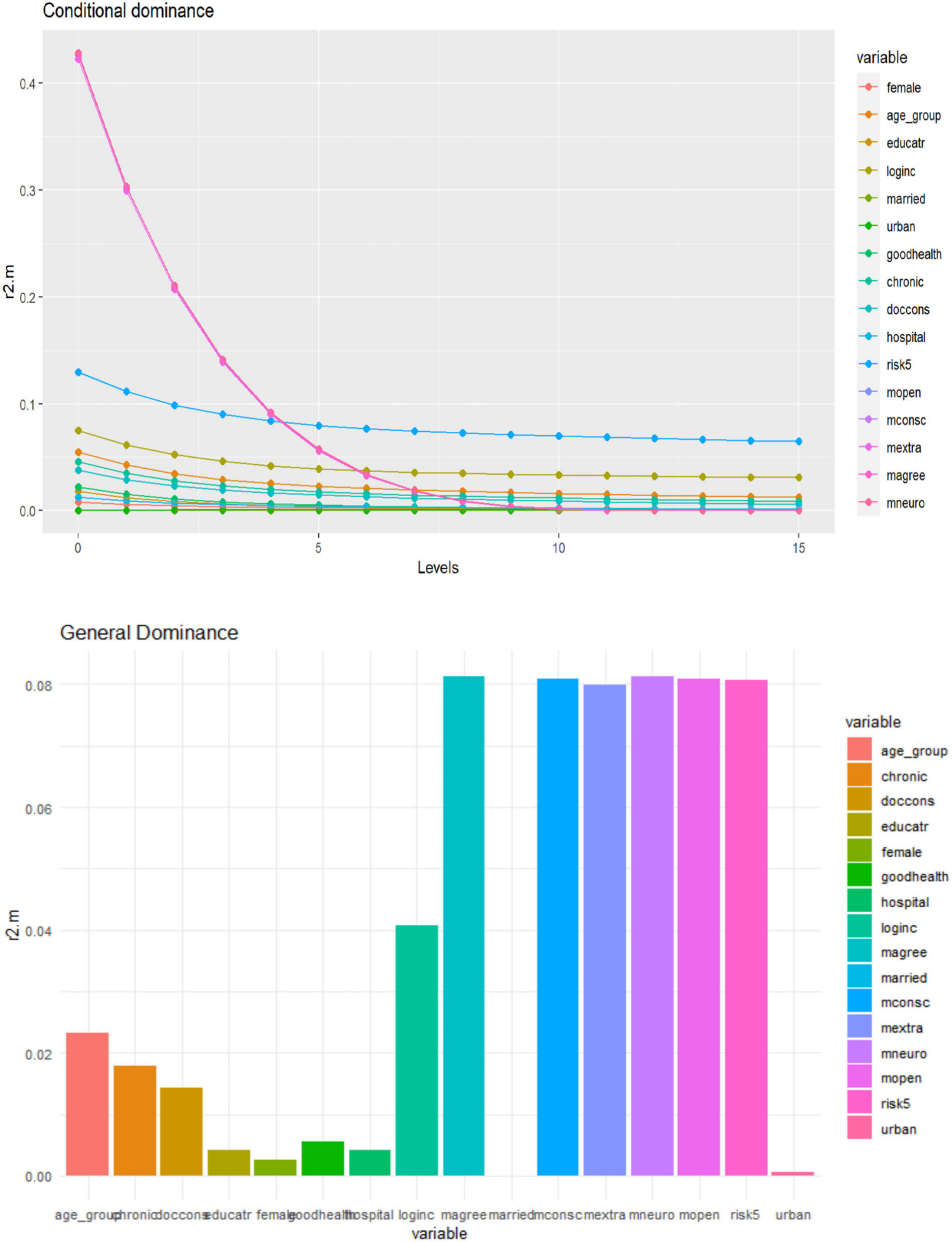
Conditional and general dominance for choice of voluntary deductibles. Swiss Household Panel (SHP), own calculations. The figure shows the contributions of predictors by levels (conditional dominance) and the average contribution (general dominance) of the predictors for the choice of deductibles, using McFadden *R*^2^ as the analog of *R*^2^. Conditional dominance statistics and general dominance statistics are displayed on the vertical axes. High values correspond to high levels of contributions, thus being more dominant across all model sizes [for more information, see (41)].

**Figure 5:**
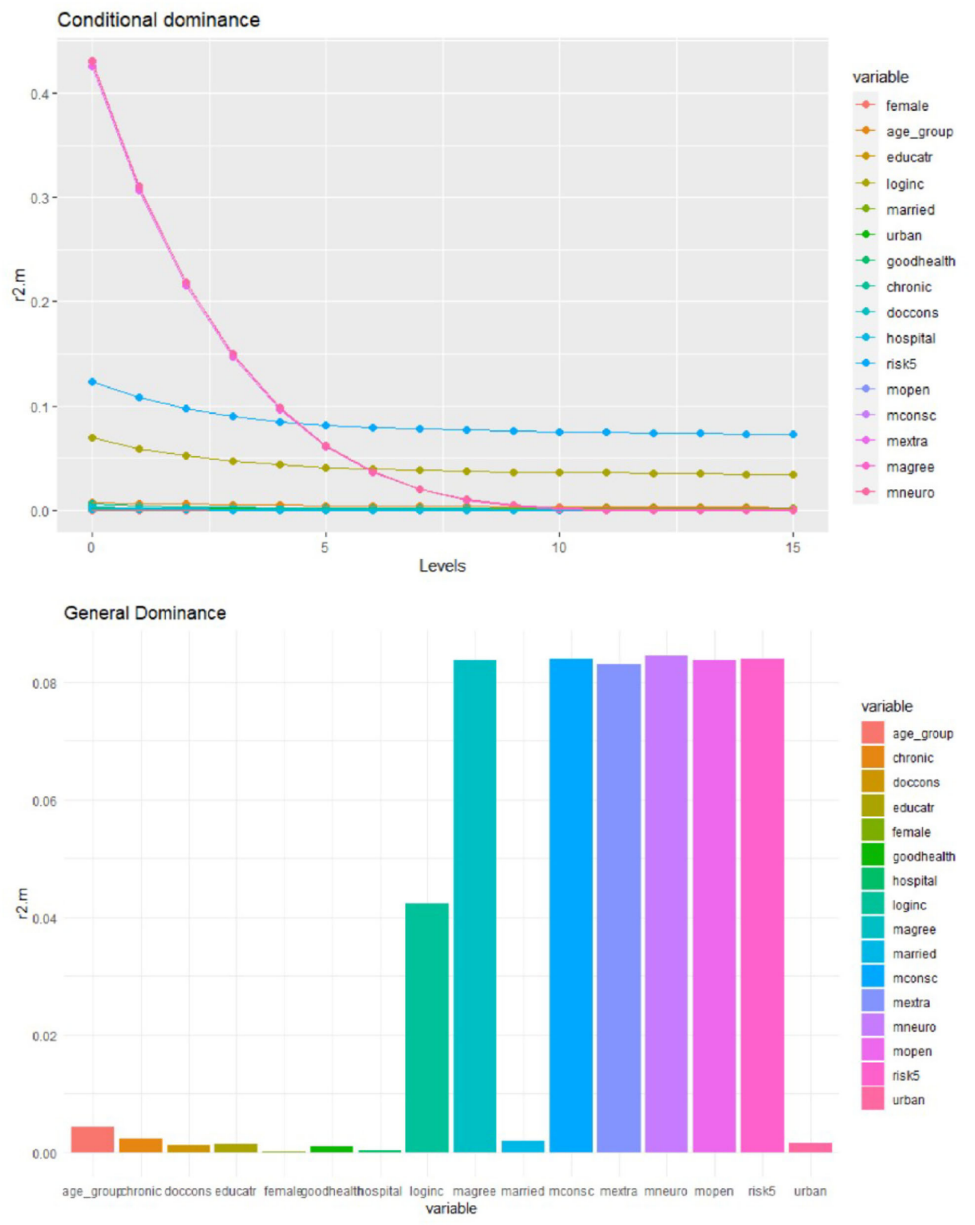
Conditional and general dominance for choice of alternative health plan. Swiss Household Panel (SHP), own calculations. The figure shows the contributions of predictors by levels (conditional dominance) and the average contribution (general dominance) of the predictors for the choice of alternative health plan, using McFadden *R*^2^ as the analog of *R*^2^. Conditional dominance statistics and general dominance statistics are displayed on the vertical axes. High values correspond to high levels of contributions, thus being more dominant across all model sizes [for more information, see (41)].

**Figure 6:**
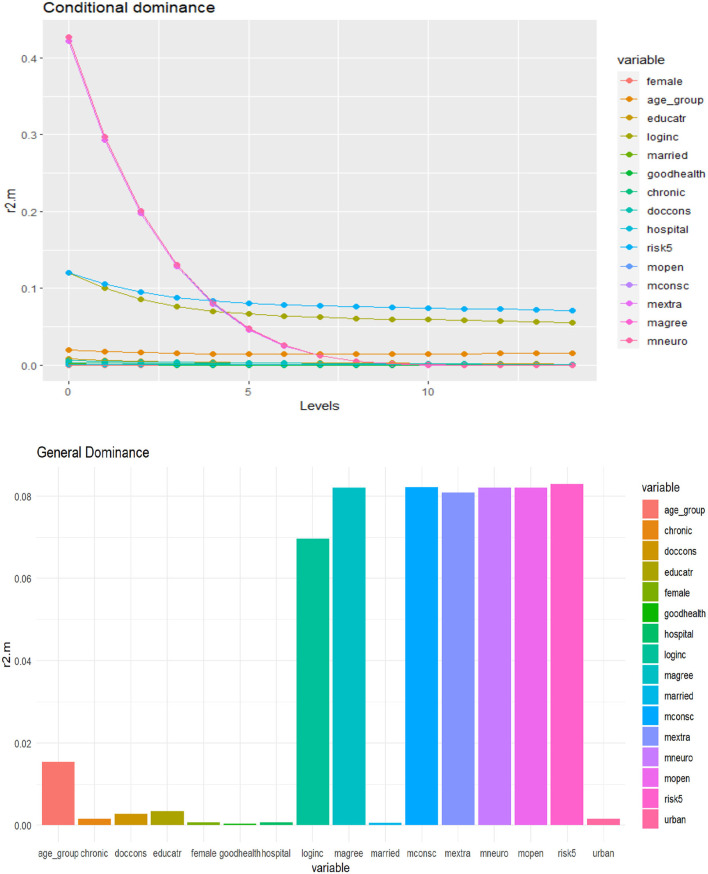
Conditional and general dominance for choice of supplementary hospital insurance. Swiss Household Panel (SHP), own calculations. The figure shows the contributions of predictors by levels (conditional dominance) and the average contribution (general dominance) of the predictors for the choice of supplementary hospital insurance, using McFadden *R*^2^ as the analog of *R*^2^. Conditional dominance statistics and general dominance statistics are displayed on the vertical axes. High values correspond to high levels of contributions, thus being more dominant across all model sizes [for more information, see (41)].

As displayed in Table 3, and Figures 4–6 personality traits and risk attitude have the largest general dominance statistics among all the predictors and for all the outcome variables. On average, every personality trait contributes more or less as much as risk attitude (roughly 8.0%) in predicting each response variable. On average, income explains 4.1, 4.2, and 7.0% of the predicted variances for choices of voluntary deductibles, managed care plans, and supplementary insurance. Age accounts for 2.3 and 1.5%, respectively, in predicting the selection of a voluntary deductible and supplementary insurance, but only 0.4% in predicting the choice of an alternative plan. Furthermore, chronic illnesses and the use of physician services explain 1.8 and 1.4%, respectively, of the predicted variance for voluntary deductible choice. However, their contributions in predicting enrollment in supplementary hospital insurance are relatively small. Predictors that are of the least importance in our models for the choice of a voluntary deductible, an alternative plan, and supplementary hospital insurance are marital status, gender, hospital stays, and subjective health status.

**Table 3:** General dominance statistics and ranking of the predictors.

**Predictors**	**Voluntary deductibles**	**Ranking**	**Alternative plans**	**Ranking**	**Private insurance**	**Ranking**
Female	0.003		0.000		0.001	
Age groups	0.023	4	0.004	5	0.015	5
Education	0.004		0.001		0.003	6
Log income	0.041	3	0.042	4	0.070	4
Married	0.000		0.002	6	0.001	
Urban	0.001		0.001		0.002	
Good health	0.006		0.001		0.000	
Chronic	0.018	5	0.002	6	0.002	
Doctor visits	0.014	6	0.001		0.003	6
Hospital stays	0.004		0.000		0.001	
Risk attitude	0.081	1	0.084	2	0.083	1
Openness	0.081	1	0.084	2	0.082	2
Conscientiousness	0.081	1	0.084	2	0.082	2
Extraversion	0.080	2	0.083	3	0.081	3
Agreeableness	0.081	1	0.084	2	0.082	2
Neuroticism	0.081	1	0.085	1	0.082	2

*Source: Swiss Household Panel (SHP), own calculations.*

*Reported numbers are the general dominance statistics for predicting the outcome variables representing choices of voluntary deductibles, alternative health plans, and supplementary private hospital insurance. Predictors with the highest values are most dominant. Only ranking levels 1–6 are reported, denoting relative importance going from higher to lower levels*.

## Discussion

This study is among the first to apply dominance analysis in logistic regression to assess the relative importance of predictors in explaining health plan choices in Switzerland. Overall, the results indicate that personality traits and attitudes toward risks play an important role in predicting health plan choices beyond common socioeconomic, demographic, and health-related factors. In the following, we will discuss our results, first providing additional intuition and explanations related to the logistic regressions for each of the three health plan choice dimensions considered in the study, then considering the new insights provided by the dominance analysis, and finally discussing the limitations of the study as well as potential directions for future research.

### Voluntary Deductibles

Our findings demonstrate that individuals who opt for a voluntary higher deductible are more likely to be in good health, have higher income, be risk-seekers, and are less likely to be female, older, and have chronic diseases. These results concur with previous findings, particularly regarding age, income, and health-related variables (6, 42–46). As people age, their health care needs, on average, increase, which leads to higher expected health care expenditures and which makes the standard deductible more attractive to reduce total out-of-pocket costs (6, 47). Moreover, in line with research from Switzerland (6) and the United States (48), we find evidence that higher income is positively associated with the probability of selecting a voluntary higher deductible. Although enrollment in health plans with a voluntary deductible leads to premium rebates, the maximum rebate does not exceed 70% of the difference between a voluntary deductible and the standard deductible (18). Hence, a person only financially benefits from the premium rebate if out-of-pocket expenditures are below the maximal rebate. In other words, higher voluntary deductibles are associated with a financial risk. If unexpected health events occur, people with a higher income can bear more financial risks and are more capable of covering a high deductible than those with a lower income.

Women are more likely to choose the lowest (standard) deductible compared to men. One possible explanation is that women are generally more risk-averse than men (47, 49, 50). Another reason is that women tend to use more health care services than men [see, e.g., (51)], although both aspects are at least partly controlled for in our model. The remaining differences could pertain to differences in health (insurance) literacy and preferences for insurance coverage, in general. As for risk attitudes, previous work showed that risk preferences are a key determinant of the decision on voluntary deductibles in the Dutch health system, which is similar to the Swiss system (47, 52). Our study confirmed this finding and revealed that risk-loving individuals tend to prefer voluntary deductibles to the standard deductible. Regarding the health-related variables, individuals in good health and without chronic conditions may expect low levels of health care utilization, which is associated with lower out-of-pocket expenses (6, 42–45). Therefore, they tend to choose voluntary deductibles in exchange for a smaller monthly premium (42–45). This also explains why individuals who did not use health care in the previous year are more likely to opt for voluntary deductibles (6, 42).

Overall, our findings with respect to deductible choice may imply the presence of selection effects and moral hazard in choosing between the standard and a voluntary deductible, as suggested by previous research on voluntary insurance selection in the Netherlands (53, 54).

### Alternative Health Plans

Regarding the choice of alternative health plans, the logistic regression results indicate that personality factors and family circumstances, rather than health-related variables, are significantly associated with opting for such a plan. More specifically, individuals who opt for an alternative health plan are more likely to be younger, married, live in a rural setting, be more extravert, and less neurotic. Regarding civil status, married people may be more concerned about budget constraints than unmarried people, and thus they may be more likely to favor a managed care plan, which offers premium rebates. On the other hand, they may also be more settled in their current place of residence, making the family doctor model a suitable and attractive alternative, which is consistent with the findings for individuals living in a rural vs. an urban setting (6). For age, recent work from Switzerland demonstrated similar results (6, 55–57). Increasing age is typically associated with higher health care needs, and thus individuals in older age groups are more likely to enroll in the more flexible standard plan, which guarantees free choice of health care providers.

In the logistic regressions, the primary link found between personality traits and choices of health plans is the role of extraversion and neuroticism in the enrollment in an alternative health plan. The likelihood of choosing such a plan is positively related to extraversion, and negatively associated with neuroticism. An individual who rates high on extraversion tends to be more assertive and sociable/outgoing, while a person who rates low on neuroticism is generally more emotionally stable and resilient. These characteristics may be associated with more thorough information-seeking and more rational decision-making, and the possible recognition that specific managed care plans may impose less restrictions on the choice of providers than otherwise suspected. The two traits might also interact with other choice predictors. For example, there is evidence that personality traits, extraversion and neuroticism in particular, are related to health care utilization (16, 58–60) and self-perceived health (61). These associations may further influence individuals' choice between the standard and a managed care plan because of prevailing access differences.

### Supplementary Private Hospital Insurance

With respect to the factors related to the subscription to supplementary hospital insurance, our findings show a similar pattern to the results from a previous study using Swiss data (62). Both the previous research and our work demonstrate that women, individuals who are older, with higher income, higher education, and relatively poor health, are more likely to buy supplementary hospital insurance (62). As people age, they might use more inpatient care, incentivizing the elderly to buy private hospital insurance. Women are more likely to purchase private hospital insurance than men. Similar to deductible choice, one reason could be that women are, on average, more risk-averse than men (47). Besides, women at certain ages, for example, in their reproductive years, might require hospital care due to fertility-related needs. The likelihood of purchasing supplementary hospital insurance also increases as education and income rise. Individuals with higher income may value greater benefit generosity, such as the free choice of specialists and a private ward in the hospital, and thus they are willing to pay for this kind of coverage. Regarding education, this might result from the interplay with income, but it could also be related to general differences in treatment preferences between high- and low-educated individuals. Compared to mandatory basic insurance, which offers community-rated premiums, supplementary hospital insurance can be risk-rated and impose access restrictions and coverage limitations (1). Demographic and socioeconomic characteristics are essential indicators that insurers can account for when deciding whether to accept an applicant, which may also explain why statistical significance is observed in the logistic regressions.

### Discussion of Findings From the Dominance Analysis

The general dominance statistics resulting from the dominance analysis somewhat differ from the results of the logistic regressions. However, this is not very surprising since statistical significance is generally not a good measure of relative predictor importance. The dominance analysis shows that income is one of the most important predictors in estimating all three outcomes, consistent with the findings in the logistic models. Unlike the logistic regressions, the general dominance statistics also reveal that personality traits and attitudes toward taking risks on average have a high contribution to predicting health plan choices across all domains considered in this study. Personality traits and risk preferences explain ~50% of the variance for each outcome, while health-related factors and most socioeconomic and demographic factors (except for income) contribute less to the model fit. However, it should be noted that chronic health conditions and doctor visits showed relatively high importance for selecting voluntary deductibles. In contrast, age is an important predictor for both deductible choice and supplementary hospital insurance.

These findings regarding personality traits and risk attitudes are worth noting since they show the strongest predictive power in all sub-models. One possible interpretation could be that personality traits correlate with risk-taking attitudes (11, 12, 63). Previous research reveals that individuals with higher levels of extraversion and openness are more likely to take risks, while those with higher levels of neuroticism, conscientiousness, and agreeableness are less likely to take risks (12, 63–65). The association between risk preferences and personality properties was also observed by other researchers (11). Therefore, it may explain why each of the personality traits provides a similar contribution to predicting health insurance decision-making as the risk attitudes. Finally, it should be noted that this correlation between and within personality traits and risk preferences is a likely explanation for why the logistic regression models partly yield statistically insignificant coefficient estimates while these factors are found important in the dominance analysis.

### Limitations and Future Research

There are a few limitations of this study. First, the variable representing extraversion could not be constructed by CFA due to computational issues. As a pragmatic approach, we constructed the mean extraversion by averaging the three items representing this domain. This resulted in a slightly larger sample size and a broader range of values for extraversion compared with the same features for openness, conscientiousness, agreeableness, and neuroticism. The variable properties may have also resulted in a general dominance statistic for extraversion that was slightly lower than the dominance statistics for the other four personality dimensions. Second, regarding the robustness checks for the dominance analysis, we only performed a bootstrapping analysis with ten replications due to the computationally intensive method. Although the dominance results were stable in these replications, we cannot prove the stability across large-scale repeated sampling.

Third, data on personality traits were collected for 2015, whereas the insurance data were derived from 2017. Although the five traits might not change between 2015 and 2017, with the current data, we cannot draw any causal inferences between personality traits and choices of health plans, and even the time-stability of personality traits may be questioned [see (23) for a related discussion]. Lastly, with our data, we could not account for individuals' health insurance literacy in our hypothesis, although it has important implications for choices of health plans. Adjusting for insurance literacy might lead to different results. Future research should address the listed limitations and provide more insights into the relative importance of other factors that influence insurance choices, promoting efficiency of decision-making and equity in using health insurance.

## Conclusions

This study first sought to examine the associations between various individual characteristics, including socio-demographic and health-related factors, risk attitudes, the Big Five personality traits, and health plan choices among the Swiss population. Second, we assessed the relative importance of the included predictors using dominance analysis. Our findings suggest that health insurance decision-making is influenced not just by classical individual characteristics but also associated with psychological factors, which have a similar relative importance as risk attitudes.

Assessing the relative importance of predictors is useful in a theoretical sense and for practical purposes (28, 29). By comparing the relative importance of various factors in predicting health plan choices, we provide novel evidence about which factors are most associated with consumers' decision-making. Based on that, more effective policies can be developed that address consumers' circumstances to enable more targeted decision support. Such measures could include, for example, consumer guidance in assessing specific insurance products and services offered in the market. Consequently, understanding the relative importance of different characteristics in explaining health plan choices may not only help better understand choice behavior but also empower individuals to make more informed decisions. This is critical for a functioning health insurance system that aims to avoid unnecessary health care expenditures, and ultimately improve health outcomes of the population by improving the quality of health insurance decisions.

## Data Availability Statement

The data analyzed in this study is subject to the following licenses/restrictions: The dataset used for this study (Swiss Household Panel) has been obtained from the Swiss Centre of Expertise in the Social Sciences (FORS). Before obtaining the data, a data contract must be signed with FORS after registration on their website. Data preparation and analysis files can be obtained from the corresponding author. Requests to access these datasets should be directed to https://forscenter.ch/projects/swiss-household-panel/.

## Author Contributions

YL and SB participated in the planning and design of the study. YL conducted the data analysis and drafted the text. SB supervised the research at all stages and finalized the text. Both authors approved the submitted version of the text.

## Funding

This project has been funded by the University of Lucerne and the State Secretariat of Education, Research, and Innovation (SERI) in Switzerland through its project-related contributions for the Swiss Learning Health System (see https://www.slhs.ch) for the period 2017-2020.

## Conflict of Interest

The authors declare that the research was conducted in the absence of any commercial or financial relationships that could be construed as a potential conflict of interest.

## Publisher's Note

All claims expressed in this article are solely those of the authors and do not necessarily represent those of their affiliated organizations, or those of the publisher, the editors and the reviewers. Any product that may be evaluated in this article, or claim that may be made by its manufacturer, is not guaranteed or endorsed by the publisher.
