# Supplementary material for: On the Relative Importance of Different Factors Explaining Health Plan Choices: Evidence From Mandatory Health Insurance in Switzerland
Source: Front Health Serv. 2022 Apr 15;2:847486. doi: 10.3389/frhs.2022.847486 (PMC10012804; doi:10.3389/frhs.2022.847486)
Supplement: Supplementary file 1 [file Table_1.docx]

**Supplementary Material**

**Table A1. Estimation results for the logistic regression models**

|  | **(1)** | **(2)** | **(3)** |
| --- | --- | --- | --- |
| **Predictors** | **Voluntary deductibles** | **Alternative plans** | **Supplementary insurance** |
| Female | 0.761^**^ | 1.028 | 1.340^***^ |
|  | (0.0634) | (0.0790) | (0.108) |
| Age (baseline: 26-35 years) | |  |  |
| *36-45 years* | 0.500^*^ | 0.895 | 1.334 |
|  | (0.139) | (0.200) | (0.338) |
| *46-55 years* | 0.436^**^ | 0.676 | 2.066^**^ |
|  | (0.118) | (0.145) | (0.501) |
| *56-55 years* | 0.262^***^ | 0.599^*^ | 2.539^***^ |
|  | (0.0709) | (0.129) | (0.618) |
| *66+* | 0.179^***^ | 0.525^**^ | 5.148^***^ |
|  | (0.0483) | (0.113) | (1.253) |
| Education level (baseline: low level) | |  |  |
| *Intermediate* | 1.270 | 1.432^**^ | 1.256 |
|  | (0.172) | (0.176) | (0.171) |
| *High* | 1.165 | 1.270 | 1.602^**^ |
|  | (0.177) | (0.176) | (0.241) |
| Income | 1.393^***^ | 0.735^***^ | 3.635^***^ |
|  | (0.122) | (0.0575) | (0.332) |
| Married (vs. not married) | 0.967 | 1.360^***^ | 1.156 |
|  | (0.0804) | (0.103) | (0.0925) |
| Urban (vs. rural) | 0.841 | 0.774^**^ | 1.086 |
|  | (0.0747) | (0.0648) | (0.0942) |
| Good health (vs. poor health) | 1.303^*^ | 1.183 | 0.992 |
|  | (0.141) | (0.118) | (0.105) |
| Chronic (yes/no) | 0.487^***^ | 0.915 | 0.906 |
|  | (0.0394) | (0.0701) | (0.0723) |
| Doctor visits (yes/no) | 0.471^***^ | 1.131 | 1.234^*^ |
|  | (0.0512) | (0.105) | (0.122) |
| Hospital stays (yes/no) | 0.691^***^ | 0.980 | 1.126 |
|  | (0.0747) | (0.0994) | (0.117) |
| Risk attitude (baseline: risk-averse) | |  |  |
| *Moderately risk-averse* | 1.468 | 0.952 | 1.401 |
|  | (0.290) | (0.173) | (0.278) |
| *Risk-neutral* | 1.423 | 0.945 | 1.401 |
|  | (0.266) | (0.161) | (0.263) |
| *Moderately risk-seeking* | 1.714^**^ | 0.933 | 1.283 |
|  | (0.327) | (0.162) | (0.244) |
| *Risk-seeking* | 1.851^**^ | 0.900 | 1.227 |
|  | (0.379) | (0.168) | (0.251) |
| Personality traits |  |  |  |
| *Openness* | 0.985 | 0.978 | 1.013 |
|  | (0.0328) | (0.0300) | (0.0328) |
| *Conscientiousness* | 1.028 | 1.106 | 1.069 |
|  | (0.0895) | (0.0881) | (0.0901) |
| *Extraversion* | 1.007 | 1.060^*^ | 1.035 |
|  | (0.0310) | (0.0303) | (0.0308) |
| *Agreeableness* | 1.129 | 1.055 | 1.050 |
|  | (0.0952) | (0.0818) | (0.0856) |
| *Neuroticism* | 0.957 | 0.897^**^ | 1.045 |
|  | (0.0366) | (0.0320) | (0.0391) |
| *N* | 3315 | 3566 | 3622 |

*Source: Swiss Household Panel (SHP), own calculations.*

*Notes*: Reported numbers are odds ratios estimated from the logistic regression models for the dependent variables indicated in the column headers and explanatory variables as shown in the table. Robust standard errors are in parentheses. For categorical regressors, the category as indicated in the table is used as a reference level. Significance levels: ^*^ *p* < 0.05, ^**^ *p* < 0.01, ^***^ *p* < 0.001.
